# Genetic polymorphism of toll-like receptors 4 gene by polymerase chain reaction-restriction fragment length polymorphisms, polymerase chain reaction-single-strand conformational polymorphism to correlate with mastitic cows

**DOI:** 10.14202/vetworld.2015.615-620

**Published:** 2015-05-15

**Authors:** Pooja H. Gupta, Nirmal A. Patel, D. N. Rank, C. G. Joshi

**Affiliations:** 1 Department of Biochemistry, B. A. College of Agriculture, Anand Agriculture University, Anand, Gujarat, India; 2 Department of Animal Genetics and Breeding, College of Veterinary Science and Animal Husbandry, Anand, Gujarat, India; 3 Department of Animal Biotechnology, College of Veterinary Science and Animal Husbandry, Anand, Gujarat, India

**Keywords:** cattle, mastitis, restriction fragment length polymorphisms, single-strand conformational polymorphism, somatic cell score, toll-like receptors 4 gene

## Abstract

**Aim:**

An attempt has been made to study the toll-like receptors 4 (TLR4) gene polymorphism from cattle DNA to correlate with mastitis cows.

**Materials and Methods:**

In present investigation, two fragments of TLR4 gene named T4CRBR1 and T4CRBR2 of a 316 bp and 382 bp were amplified by polymerase chain reaction (PCR), respectively from Kankrej (22) and Triple cross (24) cattle. The genetic polymorphisms in the two populations were detected by a single-strand conformational polymorphism in the first locus and by digesting the fragments with restriction endonuclease *Alu* I in the second one.

**Results:**

Results showed that both alleles (A and B) of two loci were found in all the two populations and the value of polymorphism information content indicated that these were highly polymorphic. Statistical results of χ^2^ test indicated that two polymorphism sites in the two populations fit with Hardy–Weinberg equilibrium (p<0.05). Meanwhile, the effect of polymorphism of TLR4 gene on the somatic cell score (SCS) indicated the cattle with allele a in T4CRBR1 showed lower SCS than that of allele B (p<0.05). Thus, the allele A might play an important role in mastitis resistance in cows.

**Conclusion:**

The relationship between the bovine mastitis trait and the polymorphism of TLR4 gene indicated that the bovine TLR4 gene may play an important role in mastitis resistance.

## Introduction

Mastitis is defined as an inflammatory reaction of the parenchyma of the mammary glands to bacterial, chemical, thermal or mechanical injury regardless of the cause and is characterized by a range of physical, chemical and usually bacteriological changes in the milk and pathological changes in the glandular tissue. Today, mastitis is considered to be a multifactorial disease, closely related to the production system and environment that the cows are kept in.

Consistently high somatic cell count (SCC) levels in a herd are usually a sign of high levels of subclinical mastitis. The most significant abnormality of the milk in subclinical mastitis is the increase in the SCC [1]. The increased leukocyte count is, in almost all cases, a reaction of tissue injury and is preceded by changes in the milk which are the direct result of damage to the tissue [2].

Many genes associated with mastitis, such as the genes of major histocompatibility complex (*MHC*), β-defensin 5, lactoferrin (LF), lysozyme, and lysostaphin, have been researched and mastitis resistant cDNA library has been constructed [3]. The bovine toll-like receptors 4 (TLR4) gene has been studied in recent years. These evolutionary conserved receptors recognize a great variety of PAMPs and consequently contribute directly to the inflammatory response [4]. For example, TLR-4 is able to recognize Gram-negative bacteria lipopolysaccharide (endotoxin) such as *Escherichia coli* and *Klebsiella*, cell wall components of other important bacteria and fungi such as *Mycobacterium tuberculosis, Aspergillus fumigatus, Cryptococcus neoformans e Candida albicans*, as well as cellular stress components, such as heat shock proteins, fibrinogen, among others [5]. Bovine mRNA of TLR4 gene was isolated by real-time-polymerase chain reaction (RT-PCR) and was quantitated by RT-PCR. The result indicated that mastitis strongly increased mRNA expression, thereby suggesting that TLR4 gene might be related with mastitis [6].

The identification of CD14, TLR-2, and TLR-4 on milk fat globule membranes suggests a direct role for the mammary gland parenchyma in pathogen detection. TLR-4 recognizes the conserved lipopolysaccharide (LPS) pattern of Gram-negative bacteria and therefore, plays an important role in the innate immune status of cows during periods of risk from intramammary infection by Gram-negative organisms [7]. It has been reported that the actual number of TLR-4 molecules involved in recognition is important for the initiation of signaling that leads to activation of the innate immune response [8]. Researchers have focused on identifying more informative genetic markers to allow faster and more accurate selection of cattle resistant to mastitis [9].

Though the disease is widely studied, very few reports exist indicating study at molecular aspects of mastitis, i.e., single nucleotide polymorphisms (SNP) in mastitis resistance gene and its correlation with inflammatory response is less documented. Ogorevc *et al*. [10] developed an extensive database of candidate genes and genetic markers for mastitis related traits. Functional traits of the mammary gland have been studied using different approaches, including the QTL approach, association studies, and the candidate gene approach. Given the facts, the work was undertaken to study the genetic polymorphism in TLR 4 gene in Kankrej and Triple cross cows by PCR-restriction fragment length polymorphisms (RFLP) and PCR-single-strand conformational polymorphism (SSCP).

## Materials and Methods

### Ethical approval

This experiment was conducted by Institutional Animal Ethics Committee (IAEC) of college of Veterinary Science & Animal Husbandry, Anand Agriculture University, Anand with ethical approval No. AAU/GVC/CPCSEA-IAEC/108/2013 dated 05/10/2013.

### Sample material

An initial screening of three hundred cows of dairy herd stationed at Livestock Research Station, Anand Agricultural University, Anand was carried out for identification of mastitis infection, of which 22 Kankrej (an indigenous dairy bred) and 24 triple crossbred (Kankrej 50% × Jersey 25% × Holstein–Friesian 25% - an exotic bred) cows were found to be infected. Milk samples of all four quarters were collected aseptically in sterile wide mouth glass stopper bottles on three consecutive days. Udder was washed thoroughly with potassium permanganate solution (1:1000), and the teats were wiped with 70% ethyl alcohol prior to sampling. Quarter milk samples were subjected to cell counters using an electronic SCC (Foss, Hillerod, Denmark) and to bacteriological culture examination. On the basis of these results (184) quarters were identified as infected with subclinical mastitis [11]. Blood samples were collected from 22 Kankrej and 24 Triple cross positive animals. The research was carried out at the Department of Animal Genetics and Breeding and Department of Animal Biotechnology, College of Veterinary Science and Animal Husbandry, Anand.

### DNA extraction and PCR

Genomic DNA was extracted from the blood samples by John’s method [12] and detected by 0.8% agarose gel electrophoresis. The content of DNA was estimated by Nanodrop ND-1000 Spectrophotometer V3.5 (Nanodrop Technologies Inc; USA) and the genome DNA was diluted to 50 ng/µL. Primers based on the fragments of *TLR4* gene (GenBank accession No. DQ839566), the coreceptor-binding region 1 (T4CRBR1) and coreceptor-binding region 2 (T4CRBR2) were selected from [6] and amplified by PCR (Table-1). The PCR reactions were carried out in a total volume of 25 µL solution containing a master mix (MBI Fermentas) system with 50 ng template DNA and 1 µL each of forward and reverse primer. The reaction conditions of PCR are shown in Table-2. The fragments of PCR were detected on a 2% agarose gel.

**Table-1:** Primer sequence for T4CRBR1 and T4CRBR2 fragments of TLR4 gene.

Gene	Disease resistance loci	Primer sequence (5’-3’)	References
TLR4	T4CRBR1	F: 5’ AGGTTGACTGGTCTCTTTG 3’, R: 5’ ACAGTGGTAGAACTCATGC 3’	[6]
TLR4	T4CRBR2	F: 5’ AGACAGCATTTCACTCCCTC 3’ R: 5’ ACCACCGACACACTGATGAT 3’	[6]

TLR4=Toll-like receptors 4

**Table-2:** Optimal condition of thermal cycler PCR for 2 region of TLR 4 gene.

Cycles	Step	Temperature	Time
1	Initial denaturation	95°C	5 min
2	1. Denaturation	94°C	30 s
	2. Annealing	58°C	30 s
	3. Extension	72°C	50 s
3	Repeat cycle 2 for 35 times		
4	Final extension	72°C	10 min

PCR=Polymerase chain reaction, TLR4=Toll-like receptors 4

### Polymorphism detection

The polymorphism of T4CRBR1 was detected with PCR-SSCP. A total of 10 µL PCR product was mixed with 3 µL of SSCP loading buffer (×6) containing 0.25% bromophenol blue and 0.25% xylene cyanol, denatured at 95°C for five min followed by snap chilling on ice for 3 min and kept on ice (at 4°C) until loaded in the gel. PCR products were subjected to SSCP on 6% non-denaturing polyacrylamide gel electrophoresis (PAGE) at 5 watts and 4°C for detection of mutations. A thermostatically controlled refrigerated circulator was used to maintain constant temperature (4°C) of the gels. The gels were run in the following conditions: 250 V, 40 mA, 10 min (pre electrophoresis), and 150 V, 24 mA, for 8 h followed by staining with silver stain and photographed by gel documentation system (GS-800, Calibrated Densitometer). The polymorphism of T4CRBR2 was detected by RFLP. The product of PCR with no purification was digested in a total of 10 µL reaction containing 1 × buffer L, 4 U *Alu* I (TaKaRa, Dalian, China), 300 ng PCR products at constant temperature (37°C) for 10 h. The products digested were electrophoresed on 12% polyacrylamide gel, which were treated at the following conditions: 200 V, 32 mA, 2.5 h. The gels were then stained by submerging the gel in 0.1% ethidium bromide solution for 30 min. The patterns of DNA bands were visualized under UV and documented with the gel documentation system (GS-800, Calibrated Densitometer).

### Traits and statistical analysis

Genotypic and allelic frequencies were calculated using the POPGENE software (ver. 1.31). The Hardy-Weinberg equilibrium of the mutation was determined by *χ*^2^ test. Traits of interest are SCC on test day, which was measured using a Foss electronic SCC in Amul dairy Anand, then converted into the somatic cell score (SCS) [13] (SCS = log [SCC/(100-000)+3]) [14]. The cows taken for investigation were of apparently healthy condition, of second to fourth lactation and of 6-9 years age. The animals belonged to single farm maintaining on the isomanagemental condition and had uniform attributable to these purity, age and health status. Thus the effects of managematic factors were ignored. Analysis of associations between the genotypes of T4CRBR1, T4CRBR2, and SCS that reflects mastitis traits was carried out with GLM procedure using SAS software (Statistical Analysis System 8.2, SAS Institute Inc.)

## Results

**Figure-1:**
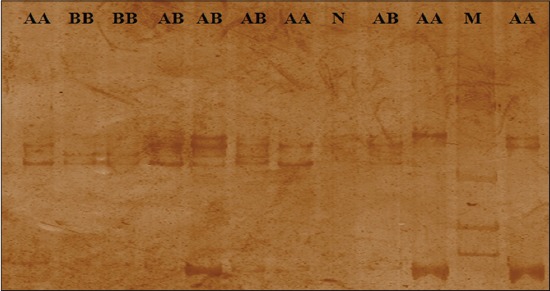
SSCP gel image of T4CRBR1 fragment of TLR4 gene, M=100bp-1031bp ladder, AA and BB=Homozygous, AB=Heterozygous.

**Figure-2:**
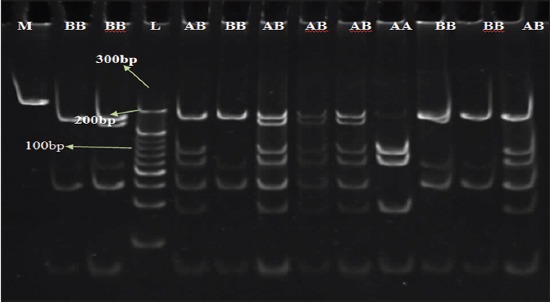
Restriction digestion gel image of T4CRBR2 fragment of TLR4 gene, L=20 bp-300 bp ladder, M=Master (PCR product of 382 bp), AA and BB=Homozygous, AB=Heterozygous.

DNA was subjected to PCR for amplification of selected regions of TLR4 gene fragment named T4CRBR1 and T4CRBR2. PCR fragment of expected size 316 bp with T4CRBR1 and 382 bp with T4CRBR2 was successfully amplified for all 46 samples. The genetic polymorphisms for the two populations in the locus T4CRBR1 was detected by SSCP (Figure-1) and the polymorphisms of T4CRBR2 were detected by RFLP (Figure-2).

SSCP analysis of loci T4CRBR1 showed three band patterns within 46 animals (Kankrej (22) and Triple cross (24)). Pattern I was observed in 20 samples (9 in Kankrej and 11 in Triple cross), pattern II was observed in 21 samples (10 in Kankrej and 11 in Triple), and pattern III in 5 samples (3 in Kankrej and 2 in Triple cross). Pattern I depicted four SSCP bands and pattern II and III showed two SSCP bands but both at a different position (Figure-1).

PCR products amplified from loci T4CRBR2, a 382 bp was further digested with restriction enzymes *Alu* I for RFLP analysis. On digestion, PCR products of 46 animals Kankrej (22) and Trile cross (24) produced three RFLP patterns. Pattern I was observed in 19 samples (11 in kankrej and 8 in Triple cross), pattern II was observed in 7 samples (4 in Kankrej and 3 in Gir), and Pattern III in 20 samples (10 in Kankrej and 10 in Triple cross). Pattern 1 depicted seven RFLP bands with base pair length of 30, 58, 80, 120, 145, 250, 270 bp, pattern II and III has four RFLP bands but both at different base pair length. Pattern II has four bands with base pair length of 30, 58 120, 145 bp, and Pattern III has 30, 80, 120, 370 bp (Figure-2).

### Genotypic and allelic frequencies

In the two populations, Kankrej and Triple crossbred have exhibited two alleles A and B in 3 different combinations AB, AA, BB. Allele A was detected in 21 heterozygous and 20 homozygous samples out of 46 samples for the region of T4CRBR1 in TLR4 gene. Allele B was detected in 21 heterozygous and 4 homozygous samples out of 46 samples for the region of T4CRBR2 in TLR4 gene. The genotypic and allelic frequencies of two loci are shown in Table-3. Genotypes at both the loci within a bred were distributed as per Hardy-Weinberg equilibrium.

**Table-3:** Genotypic and allelic frequencies for 2 breeds of cow for TLR4 gene.

Locus	Breeds	No	Genotypic frequency			Allelic frequency		*χ*^2^
	
			AA	AB	BB	A	B	
T4CRBR 1	Kankrej	22	0.4545	0.4090	0.1363	0.654	0.341	0.177
	Triple cross	24	0.4583	0.5000	0.0416	0.708	0.292	1.062
T4CRBR 2	Kankrej	22	0.1363	0.5000	0.3636	0.386	0.614	0.065
	Triple cross	24	0.1250	0.4583	0.4166	0.354	0.646	0.000

TLR4=Toll-like receptors 4

In the two populations, allele A was the predominant allele of T4CRBR1 locus, whereas allele *B* was the predominant allele of T4CRBR2 locus. The value of AB genotypic frequency was maximum in both loci, BB genotypic frequency was minimum in T4CRBR1 locus, but AA genotypic frequency was minimum in T4CRBR2 locus.

### Genetic character in the two populations

Table-4 shows the value of heterozygosity (H), effective number of alleles (Ne), polymorphism information contents (PIC), and *χ*^2^ value for two breds, Kankrej, and Triple crossbred. The PIC ranged from 0.25 to 0.50, which indicated that both loci, i.e., T4CRBR1 and T4CRBR2 of the two populations were moderate polymorphic. The value of PIC and H of Triple crossbred in second loci was higher than that of the Kankrej, which implied that the polymorphism and the genetic variation in the two loci of Triple cross were higher than that of the Kankrej. The result of Hardy–Weinberg equilibrium indicated that two polymorphic sites in the both populations confirmed with Hardy–Weinberg equilibrium (p<0.05).

**Table-4:** Data of H, Ne, PIC of 2 loci of TLR4 gene.

Locus	Breeds	PIC	H	Ne
T4CRBR1	Kankrej	0.3398	0.4339	1.7664
	Triple cross	0.3398	0.4339	1.7041
T4CRBR2	Kankrej	0.3278	0.4132	1.7664
	Triple cross	0.3528	0.4575	1.8432

The number of significant (p<0.05) linkage disequilibrium, H=heterozygosities, Ne=Effective number of alleles, PIC=Polymorphism information contents

The relationship between bovine mastitis trait and the polymorphism of TLR4 gene: The analysis of variance on SCS was calculated using the model with genetic marker effect. Table-5 shows that4e genotypic effect of T4CRBR2 considerably affect the SCS. Meanwhile, the effect of different breed type on SCS was analyzed. The value of SCS of Triple crossbred was significantly higher than that of the Kankrej. Hence, it was concluded that Crossbreed cattle were easier to be infected with mastitis than pure breeds (Table-6). However, for locus T4CRBR1, the cattle with the AA genotype (4.3) and AB genotype (3.9) showed lower SCS in comparison to the cattle with the BB genotype (6.0) (Table-7). The result indicated that the cattle with the AA and AB genotypes were less prone to be infected with mastitis than the cattle with the BB genotype. In other words, allele A might be the beneficial allele for mastitis resistance.

**Table-5:** Effects of a genotypic factor on somatic cell score.

Factors	Genotype of T4CRBR1	Genotype of T4CRBR2
F value	4.7	5.0

**Table-6:** Effects of breeds on somatic cell score.

Breeds	Kankrej	Triple cross
LSM	3.42	5.29

LSM=Least squares mean

**Table-7:** Effects of different genotypes on somatic cell score.

Genotype	Locus	AA	AB	BB
LSM	T4CRBR1	4.3	3.9	6.0
	T4CRBR2	5.1	5.7	4.3

LSM=Least squares mean

## Discussion

Polymorphisms in genes encoding receptors associated with the innate immune system are likely to contribute to the overall variation in the resistance or susceptibility to mastitis in dairy cattle. Present study was carried out to find out the polymorphism at two loci within TLR4 gene where the polymorphism is reported by [6]. Results suggest that heterozygous were predominant for both loci. The result of Hardy–Weinberg equilibrium for the two loci in the two populations agreed with Hardy–Weinberg equilibrium and showed similar results with [6]. As the present study was carried out on the pure breed and Triple cross cattle and Triple crossbred may have highly polymorphic loci due to cross breeding, it might have distorted the equilibrium. Present data with SCS was well matched with the work done by [6] in relation to genotypic data only. Thus from our work, SCS were significantly affected by both the type of breeds (p<0.05) and by different genotypes (p<0.05). Further, the effect of the TLR4 polymorphism on SCS in milk was analyzed and showed SCS reflect the degree of mastitis [15]. An association study between the SNP and SCS in the three breeds showed that the SCS of individuals with a CC genotype was significantly lower than that of the TT genotype [16].

Several reports are available for TLR4 gene and mastitis, but some of them showed a positive correlation of TLR4 gene with Mastitis. Mesquita [17] studied Brazilian Holsteins for TLR4 polymorphism indicated that animals with combined genotypes AACCCC, GGTCGG, and GACCGC presented the lowest SCS and have the potential to be applied as molecular markers for assisted animal selection to improve milk quality. Carvajal *et al*. [18] evaluated three SNPs contained in (TLR4) and lactoferrin genes of Chilean dairy cattle associated with mastitis traits: TLR4 P-226, TLR4 2021, and LF P-28. Results showed the TT genotype of TLR4 2021 was significantly associated with the healthy condition, but no associations with SCS were evident. Noori *et al*. [19] showed the B allele of the SNP in T4CRBR2 of the TLR4 gene was associated with higher 305-day milk yield and breeding value for milk yield, and lower fat percentage and lower SCS, as compared with allele A. The B allele frequency was higher and the distribution of genotypes was not in Hardy-Weinberg equilibrium in the overall population. A study by Beecher [20] indicated an association of E3+2021 polymorphism in TLR4 gene with milk fat and protein percentage in late lactation in Holstein–Friesian, Jersey, Norwegian Red, Montbeliarde, and Holstein–Friesian × Jersey cows, but not in Holstein–Friesian bulls. Gulhane [21] observed significant (p≤0.05) difference in the genotypic frequencies of the two genotypes in healthy and mastitis Murrah buffaloes. The frequency of AA genotype was significantly higher (p≤0.05) in healthy animals and indicated the association of AA genotype with resistance to mastitis. Mitra [22] studied TLR-4 gene of *Murrah buffaloes* and was found highly polymorphic with AA, AB, and BB genotypes as revealed by PCR-RFLP analysis using Dra I, Hae III, and Hinf I REs. Nucleotide sequencing of the amplified fragment of TLR-4 gene showed twelve SNPs with six non-synonymous SNPs resulting in a change in amino acids.

Based on the above studies on the role of TLR4 in pathogen recognition and subsequent initiation of the inflammatory and immune response, and on differential expression of the gene during mastitis, TLR4 has been proposed as a candidate for increasing mastitis resistance in breeding programs [10]. Hence, a thorough investigation of bovine TLR4 gene is of immense value. The relationship between the bovine mastitis trait and the polymorphism of TLR4 gene indicated that the bovine TLR4 gene may play an important role in mastitis resistance, which is consistent with the other reports [23].

## Conclusion

Overall the results are concluded that Triple crossbred have high number of SCC as compared to Kankrej, which indicate that the SCS was significantly affected by the type of breeds. The indigenous cattle are said to be more resistant to mastitis than crossbred cows with the exotic breed. Furthermore, *Bos indicus* are believed to be more disease resistant than *Bos taurus*. The genotype AA of T4CRBR1 was related to mastitis resistance so allele ‘*a’* may be the beneficial allele for mastitis resistance, whereas genotype BB was related to mastitis susceptibility. It was easy to conclude that cow TLR4 gene may play an important role in mastitis resistance.

## Authors’ Contributions

PHG carried out molecular work for screening of resistant and susceptible mastitic cows, NAP performed initial screening of infected cows; DNR guided the research work, and CGJ helped in the analysis of data. All authors participated in draft and revision of the manuscript. All authors read and approved the final manuscript.
